# Complete Genome Sequence of a Novel Monopartite *Mastrevirus*, Soybean Geminivirus B, Isolated from Soybean (*Glycine max* (L.) Merrill)

**DOI:** 10.3390/plants11131768

**Published:** 2022-07-03

**Authors:** Hoseong Choi, Yeonhwa Jo, Jinsung Hong, Hyunjung Chung, Sooyeon Choi, Sangmin Kim, Jeonghun Lee, Sanghyun Moh, Bongchoon Lee, Won Kyong Cho

**Affiliations:** 1 Plant Genomics and Breeding Institute, Seoul National University, Seoul 08826, Korea; bioplanths@gmail.com; 2 College of Biotechnology and Bioengineering, Sungkyunkwan University, Seoburo 2066, Suwon 16419, Korea; yeonhwajo@gmail.com; 3 Department of Applied Biology, Kangwon National University, Chuncheon 24341, Korea; jinsunghong@kangwon.ac.kr; 4 Crop Foundation Division, National Institute of Crop Science, Rural Development Administration, Wanju 55365, Korea; chunghyunjung@korea.kr (H.C.); choisy99@korea.kr (S.C.); kimsangmin@korea.kr (S.K.); 5 Plant Cell Research Institute of BIO-FD&C Co., Ltd., Incheon 21990, Korea; jhlee@biofdnc.com (J.L.); shmoh@biofdnc.com (S.M.)

**Keywords:** genome, geminivirus, *Mastrevirus*, soybean, soybean geminivirus B

## Abstract

Soybean is one of the most important crops in Korea. To identify the viruses infecting soybean, we conducted RNA sequencing with samples displaying symptoms of viral disease. A contig displaying sequence similarity to the known *Geminivirus* was identified. A polymerase chain reaction (PCR) using two different pairs of back-to-back primers and rolling circle amplification (RCA) confirmed the complete genome of a novel virus named soybean geminivirus B (SGVB), consisting of a circular monopartite DNA genome measuring 2616 nucleotides (nt) in length. SGVB contains four open reading frames (ORFs) and three intergenic regions (IRs). IR1 includes a nonanucleotide origin of replication in the stem-loop structure. Phylogenetic and BLAST analyses demonstrated that SGVB could be a novel virus belonging to the genus *Mastrevirus* in the family *Geminiviridae*. We generated infectious clones for SGVB by adding a copy of the IR1 region of SGVB, comparing the V-*ori* in addition to the full-length genome of SGVB. Using the infectious clones, we observed chlorosis and leaf curling with a latent infection in the inoculated *Nicotiana benthamiana* plants, while none of the inoculated soybean plants showed any visible symptoms of disease. This study provides the complete genome sequence and infectious clones of a novel *Mastrevirus* referred to as SGVB from soybean in Korea.

## 1. Introduction

Geminiviruses are plant viruses that cause severe viral diseases in a wide range of crop plants, resulting in massive reductions in crop production [1]. They are members of the family *Geminiviridae*, and their genomes consist of single-stranded circular DNA that encodes genes in both directions from an origin of replication [2]. To date, more than 360 species in the nine genera of the family *Geminiviridae* have been reported [3].

Soybean (*Glycine max* (L.) Merrill), a member of the subfamily Fabaceae, is a kind of annual self-pollinated diploid legume [4]. It is one of the most important crops in the world and a major source of vegetable oil and protein in many Asian countries. Soybean is susceptible to a large number of plant viruses [5], many of which are RNA viruses. To date, only a few DNA viruses infecting soybean have been identified [6]. For example, three different begomoviruses in the family *Geminiviridae*, including the bean golden mosaic virus (BGMV), the sida micrantha mosaic virus (SiMoV), and the okra mottle virus (OMoV), have been identified in soybean in central Brazil [6].

Recently, several viruses infecting soybean have been identified. Of these, the soybean mosaic virus (SMV), the soybean yellow mottle mosaic virus (SYMMV), and the soybean yellow common mosaic virus (SYCMV) have been identified as major viruses infecting soybean in Korea [7]. Other viruses infecting soybean in Korea include the peanut mottle virus (PeMV), the soybean dwarf virus, the clover yellow vein virus, the bean common mosaic virus, the peanut stunt virus, the tomato spotted wilt virus, and the bean common mosaic necrosis virus [8,9,10,11,12,13,14]. Although various RNA viruses infecting soybean have been identified, none of the DNA viruses infecting soybean have been reported in Korea.

Recently, we carried out RNA-sequencing to identify viruses infecting soybean. We found a contig showing sequence similarity to the known chickpea chlorotic dwarf virus (CpCDV), a kind of geminivirus, from the soybean transcriptome. Here, we determined the full-length genome of the novel mastrevirus referred to as soybean geminivirus B (SGVB) and generated infectious clones for SGVB. Using the infectious clones, we examined the disease symptoms caused by SGVB in three different soybean cultivars and *Nicotiana benthamiana* plants through agroinoculation.

## 2. Materials and Methods

### 2.1. Soybean Samples

We collected a total of 16 soybean samples grown in the experimental field of Chungcheongbuk-do Agricultural Research and Extension Services on 28 July 2016. All soybean leaf samples displayed viral disease symptoms, including mosaic, mottling, and yellowing. The collected leaves were immediately frozen in the presence of liquid nitrogen.

### 2.2. Library Preparation and RNA-Sequencing

Individual frozen leaf tissues were ground in a mortar with a pestle. The same amount of fine powder from each sample of leaf tissue was pooled and subjected to total RNA extraction using the RNeasy Microarray Tissue Mini Kit, according to the manufacturer’s instructions (Qiagen, Hilden, Germany). The total RNA was used for the RNA-sequencing library preparation, which was carried out using the TruSeq RNA Library Preparation Kit v2 (Illumina, San Diego, CA, USA), as described previously [14]. The library was paired-end sequenced by Illumina’s HiSeq2000 system (Macrogen, Seoul, Korea).

### 2.3. Bioinformatics Analyses to Identify Virus-Associated Contigs

The obtained raw sequence reads were *de novo* assembled by the Trinity program using default parameters [15]. The obtained contigs were subjected to a BLAST search, with E-value 1 × 10^−10^ as a cutoff, against the reference viral database obtained from the National Center for Biotechnology Information (NCBI) (https://www.ncbi.nlm.nih.gov/genome/viruses/, accessed on 8 June 2022). The obtained virus-associated contigs were again subjected to a BLAST search against the NCBI nucleotide database to remove virus-like sequences from the host. Finally, virus-associated contigs were obtained.

### 2.4. Complete Genome Sequencing of SGVB

To acquire the complete genome sequence of the identified DNA virus named SGVB, we extracted total DNA from the same soybean plant used for RNA-sequencing, using the DNeasy Plant Mini Kit according to the manufacturer’s instructions (Qiagen, Hilden, Germany).

We conducted a polymerase chain reaction (PCR) to obtain the complete genome of SGVB using the two different pairs of back-to-back primers as follows. Firstly, we designed a primer pair (SGVB1_for 5′–ATGGTGGGACCCTTTAAAGGTAAC–3′ and SGVB1_rev 5′–TTATTGATTACCAACGGACTTGAAGTACA–3) based on a partial sequence of SGVB derived from RNA-sequencing. The PCR was conducted with SGVB1_for and SGVB1_rev primers using Phusion high-fidelity DNA polymerase (NEB, Ipswich, U.S.A.). The amplified PCR product was cloned into pGEM-T easy vector (Promega, Madison, U.S.A.), and the partial SGVB sequence was confirmed. Next, we designed the second primer pair (SGVB2_for 5′–AGTCAACGCTCCATCAAGGT–3′ and SGVB2_rev 5′– CAGATCCCGTTGGGAGTTAA–3). We amplified the full-length genome sequence of SGVB by PCR with SGVB2_for and SGVB_2 rev primers using GXL polymerase (Takara, Shiga, Japan). The amplified PCR product was cloned into pGEM-T easy vector (Promega) and sequenced by Sanger-sequencing (Macrogen).

In addition, circular viral DNA was amplified by the rolling circle amplification (RCA) method using Φ29 DNA polymerase (New England Biolabs Inc., Ipswich, MA, USA). We digested the RCA product with *Bam*HI, resulting in a ~2.6 kb fragment. The digested DNA fragment was eluted from the agarose gel using the QIAquick Gel Extraction Kit (Qiagen). After that, the DNA fragment was further cloned in *Bam*HI-restricted pGEM-3zf (+) (Promega). The full-length clones from the RCA were sequenced using the primer walking approach (Macrogen).

Finally, we obtained a full-length genome sequence of SGVB isolate Kong measuring 2616 nt in length. The complete genome sequence of SGVB isolate Kong was deposited in GenBank under the accession number MH428830.

### 2.5. Prediction of Open Reading Frames (ORFs), Conserved Domains, and Stem-Loop Structure

The ORFs for the complete genome of SGVB were predicted by ORFfinder (https://www.ncbi.nlm.nih.gov/orffinder/, accessed on 8 June 2022). The conserved domains in SGVB were predicted by the SMART program (https://smart.embl.de/, accessed on 8 June 2022) [16]. The stem-loop structure in the intergenic region (IR) of SGVB was predicted by the mfold web server (http://www.unafold.org/mfold/applications/dna-folding-form.php, accessed on 8 June 2022) [17].

### 2.6. Pairwise Sequence Alignment and Visualization

The full-length genome of SGVB and its homologous CpCDV isolate, CpCDV_H_SD_SD180_2013, were pairwise aligned using ClustalW, implemented in the TCOFFEE program (http://tcoffee.crg.cat/apps/tcoffee/index.html, accessed on 8 June 2022) [18]. The aligned sequences were visualized by ESPript 3 (http://espript.ibcp.fr/ESPript/cgi-bin/ESPript.cgi, accessed on 8 June 2022) [19].

### 2.7. Construction of Phylogenetic Trees

The full-length genome of SGVB and the replicase protein (C1) of SGVB were subjected to a BLASTN search to find homologous viral genomes and proteins, respectively. The obtained sequences were aligned by MAFFT [20] and trimmed by the trimAl program [21]. The trimmed sequences were subjected to the construction of phylogenetic trees with the MEGA7 program, using the maximum likelihood (ML) method with 1000 bootstrap values [22].

### 2.8. Construction of Full-Length Infectious Clones for SGVB

We generated infectious clones for SGVB by constructing the full-length viral DNA of SGVB. We inserted the sequences of IR1 of SGVB into the full-length genome sequence of SGVB, resulting in two copies of the IR1 sequences. Both the IR1 + SGVB1 genome sequences and the pCAMBIA2300 vector were digested by *Sma*I. The digested viral DNA fragment was ligated to the slightly modified pCAMBIA2300 vector. The generated construct was transformed into *Escherichia coli* strain TOP10F by heat shock. The plasmid DNA was purified and transformed into *Agrobacterium tumefaciens* strain GV3101 by heat shock.

To amplify the IR of SGVB, we carried out the PCR with two primers (LIR-F: 5′–GGAGGCAAGGGCATTGTGG–5′ and RepA-F: 5′–ATGCCTCCACGCCCTGTTAT–3′). The PCR product was cloned into the modified pCAMBIA2300 vector treated by *Sma*I. Since we attached the restriction enzyme site (*Sma*I) to the end of the RepA-F primer, the full-length genome sequence of SGVB was ligated to the IR+pCMABIA2300 vector that was digested by *Sma*I.

### 2.9. Plant Materials, Growth Conditions, and Agroinoculation

Three different soybean cultivars, named Buseok, Cheongja 3-ho, and Taekwang, respectively, were grown in a growth chamber at 25 °C under a 16/8 h photoperiod. Plasmid DNA (10 µg) was rub-inoculated onto the fully-expanded cotyledons of soybean seedlings. At 30 d post-infection, we observed viral disease symptoms in the inoculated soybeans. We used three different plants for each soybean cultivar. Two independent experiments were conducted for the rub-inoculation.

*N. benthamiana* plants were grown in the growth chamber at 22 °C under a 14/10 h photoperiod. We agroinoculated the leaves of *N. benthamiana*. At 3 weeks post-infection, we observed viral disease symptoms in the inoculated *N. benthamiana* plants. To confirm viral accumulation in the inoculated and upper leaves of *N. benthamiana*, we extracted total genomic DNA and performed a PCR using the SGVB1_for and SGVB1_rev primers.

## 3. Results

### 3.1. Identification of a Novel DNA Virus in Soybean Using RNA-Sequencing

To identify the viruses infecting soybean, we collected 16 samples of soybean leaf displaying viral disease symptoms. Each individual soybean leaf sample was used for the RT-PCR, conducted using virus-specific primers for SMV, SYMMV, and SYCMV. All the samples were co-infected by at least two different viruses. The pooled samples were used for total RNA extraction followed by library preparation for RNA-sequencing using the HiSeq 2000 system. RNA-sequencing resulted in 4,581,738,548 bp (45,363,748 reads). De novo transcriptome assembly followed by a BLAST search against the viral database identified a total of 57 virus-associated contigs. We identified SMV, SYMMV, SYCMV, and PeMV. In addition, we found that a contig measuring 2130 nucleotides (nt) in length showed sequence similarity to the known CpCDV (GenBank NC_011058.1). The contig was again blasted against the nucleotide database in the NCBI. The contig showed sequence similarity to the CpCDV strain R isolate BF:Tan:To164:15 (GenBank KY047533.1), with a query coverage of 73% and a sequence identity of 80%, suggesting a partial sequence of a novel viral genome. We tentatively named the novel virus as soybean geminivirus B (SGVB) isolate Kong.

### 3.2. Complete Genome Sequencing of SGVB

**Figure 1:**
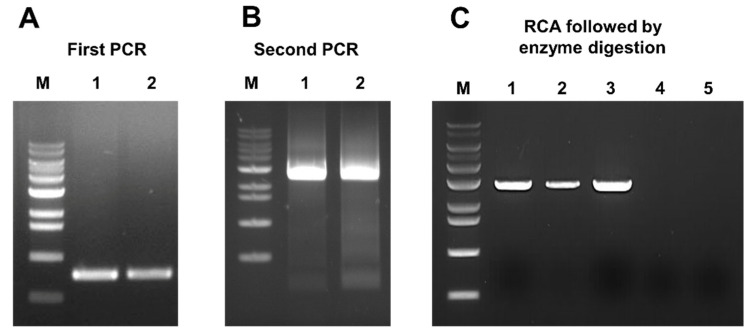
Amplification of the soybean geminivirus B (SGVB) genome by polymerase chain reaction (PCR) and rolling circle amplification (RCA) methods. (**A**) Amplification of the SGVB partial sequence by the first PCR using SGVB1_for and SGVB1_rev primers. M indicates the DNA ladder. Lines 1 and 2 indicate two independent PCR reactions. (**B**) Amplification of the linear complete SGVB genome by the second PCR using SGVB2_for and SGVB2_rev primers. M indicates the DNA ladder. Lines 1 and 2 indicate two independent PCR reactions. (**C**) Three independent SGVB genomes amplified by RCA followed by enzyme digestion with *Bam*HI (lines 1 to 3) and without enzyme digestion (line 4). Control soybean sample without SGVB infection (line 5).

The BLAST result suggested that SGVB might be a member of the family Geminiviridae with a single-stranded circular DNA genome. To obtain the complete genome sequence of SGVB, we carried out PCRs using the two different pairs of back-to-back primers. Firstly, we performed a PCR using SGVB1_for and SGVB1_rev primers to confirm the partial sequence of SGVB derived from RNA-sequencing (Figure 1A). After that, we designed new primers (SGVB2_for and SGVB2_rev) to amplify the complete genome of SGVB based on the Sanger-sequencing result. In the second PCR, we amplified approximately 2.6 kb of PCR product (Figure 1B), which was cloned and then subjected to Sanger-sequencing. In addition, RCA was conducted to amplify the circular viral DNA. Subsequently, the amplified circular DNA was digested by BamHI, resulting in linear DNA with ~2.6 kb (Figure 1C). The digested DNA was cloned and sequenced using the primer walking approach. Finally, we obtained the complete genome of SGVB measuring 2616 nt in length (GenBank MH428830).

### 3.3. Genome Organization of SGVB

**Figure 2:**
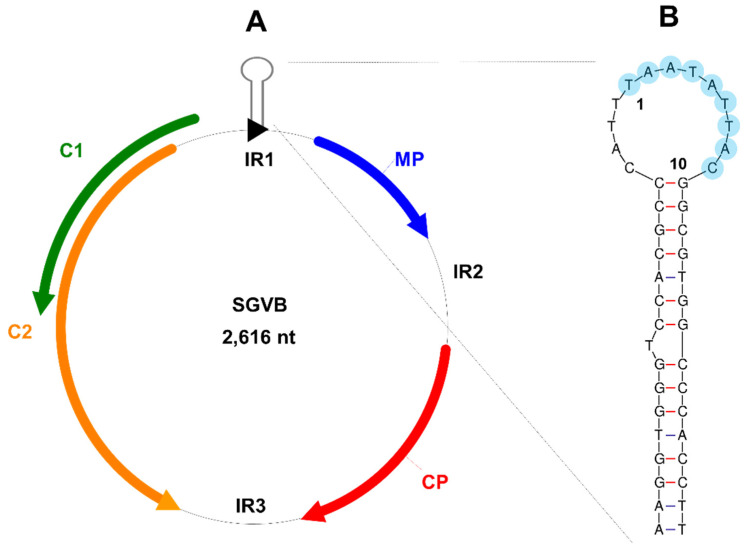
Genome organization of SGVB. (**A**) Genome organization of SGVB. Four ORFs encoded by SGVB and three IRs are depicted. The MP and CP are encoded on the virion-sense strand, while the C1 and C2 are encoded on the virion-antisense strand. MP, movement protein; CP, coat protein. (**B**) The stem-loop structure of the conserved 5′-TAATATTAC-3′ (blue circles) in the IR1 region is depicted. The number 1 indicates the virus replication origin.

The BLAST result with the complete nucleotide genome of the SGVB genome showed sequence similarity to the CpCDV isolate CpCDV_H_SD_SD180_2013 (GenBank KM229842.1), with a query coverage of 76% and a sequence identity of 80.80%. SGVB contains four ORFs encoding as movement protein (MP), coat protein (CP), and C1 and C2 proteins (Figure 2A). In addition, SGVB includes three IRs. IR1 (264 nt) is located between the C1 and MP and contains a nonanucleotide origin of replication (5′–TAATATTAC–3′) in the stem-loop structure, which is highly conserved in geminiviruses (Figure 2B). IR2 (266 nt) is located between the MP and CP, while IR3 (265 nt) is located between the C2 and CP. The MP and CP are encoded on the virion-sense strand, while the C1 and C2 proteins are encoded on the virion-antisense strand (Figure 2A). The BLASTP results showed that the SGVB MP, C1, and C2 proteins exhibited sequence similarity to the MP, RepB, and RepA proteins of CpCDV, a monopartite, single-stranded circular DNA virus in the genus *Mastrevirus* and the family *Geminiviridae* (Table 1). The SGVB CP showed sequence similarity to the CP of chickpea redleaf virus 2 in the genus *Mastrevirus* and the family *Geminiviridae*.

### 3.4. Phylogenetic Relationship of SGVB with Other Geminiviruses

**Figure 3:**
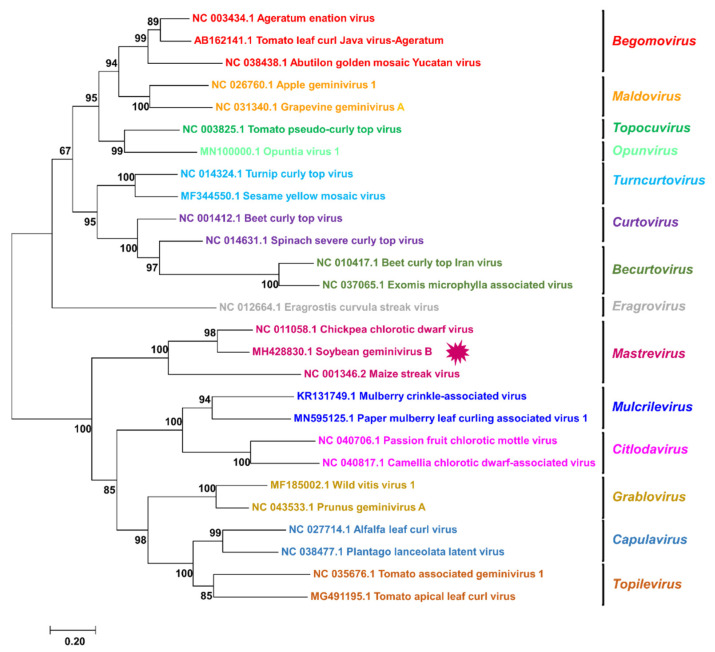
Phylogenetic relationships of SGVB with known geminiviruses. aximum likelihood (ML) phylogenetic tree based on full-length genome sequences with 1000 bootstrap replicates displays phylogenetic relationships between SGVB and 26 other geminiviruses. Bootstrap values are indicated at nodes.

**Figure 4:**
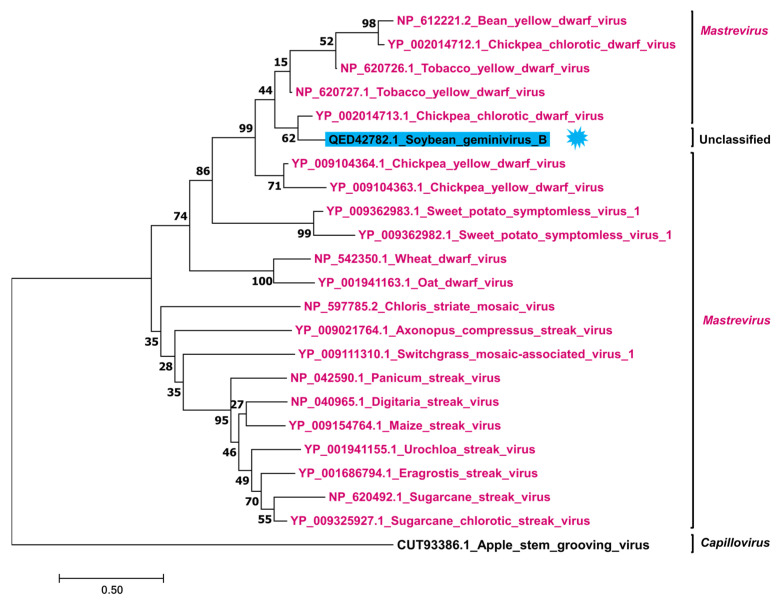
Phylogenetic relationships of SGVB with known geminiviruses. The ML phylogenetic tree based on replicase protein sequences with 1000 bootstrap replicates displays phylogenetic relationships between the SGVB genome and 21 other geminiviruses. The replicase protein of the apple stem grooving virus was used as an outgroup. Bootstrap values are indicated at nodes.

To reveal the phylogenetic relationship of SGVB with other known geminiviruses, we generated two phylogenetic trees using complete genome sequences (Figure 3) and replicase proteins (Figure 4), respectively. A phylogenetic tree based on full-length genomes showed that SGVB was closely related to CpCDV (GenBank NC_011058.1) and the maize streak virus (GenBank NC_001346.2), which are members of the genus *Mastrevirus* (Figure 3). The phylogenetic tree clearly showed that the three viruses, including SGVB in the *Mastrevirus* genus, were distinguishable from other genera. Thus, phylogenetic analyses indicated that the SGVB isolate Kong is a novel virus, which could be a putative member of the genus *Mastrevirus* in the family *Geminiviridae*.

Using the C1 protein of SGVB as a query, we identified 20 viral proteins. Interestingly, all 20 replicase-associated proteins were derived from *Mastreviruses*. The phylogenetic tree using replicase proteins revealed that SGVB showed a strong sequence similarity to two known C1 proteins of CpCDV (Figure 3). In addition, the SGVB C1 protein was closely related to the replicase protein of the tobacco yellow dwarf virus, the chickpea yellow dwarf virus, the sweet potato symptomless virus 1 (SPSV1), the wheat dwarf virus, and the oat dwarf virus.

**Figure 5:**
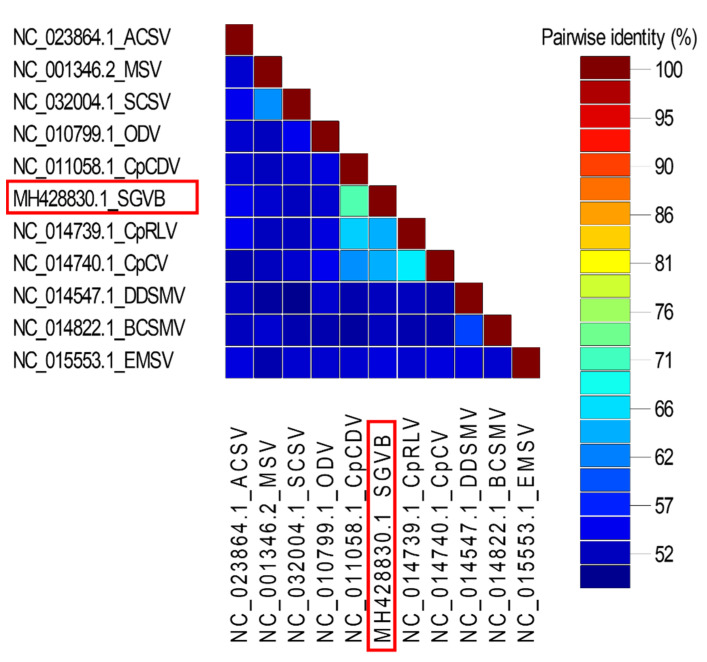
Pairwise sequence comparison of the genomes of SGVB and 10 other geminiviruses using a sequence demarcation tool. For the comparison, we selected geminiviruses showing a high sequence similarity to SGVB based on BLASTN results. Pairwise identity (%) is visualized by the different colored keys.

Next, we carried out a pairwise sequence comparison of SGVB and 10 geminiviruses. The full-length genomes of SGVB and the 10 selected geminiviruses were aligned by MAFFT and subjected to a pairwise comparison using SDT version 1.2 [23]. The nucleotide identity matrix showed that the sequence similarity of SGVB with other known viruses ranged from 52% to 71% (Figure 5). Specifically, the pairwise sequence alignment between the complete genome of SGVB and the CpCDV isolate CpCDV_H_SD_SD180_2013 showed 71.34% nucleotide identity (Figure S1). According to the criteria for species demarcation for the genus *Mastrevirus*, the full-length nucleotide sequence identity of an isolate should be less than 78%. Thus, SGVB could be a novel virus assigned to the genus *Mastrevirus*.

### 3.5. Generation of Infectious Clones for SGVB and Disease Symptoms Caused by SGVB

**Figure 6:**
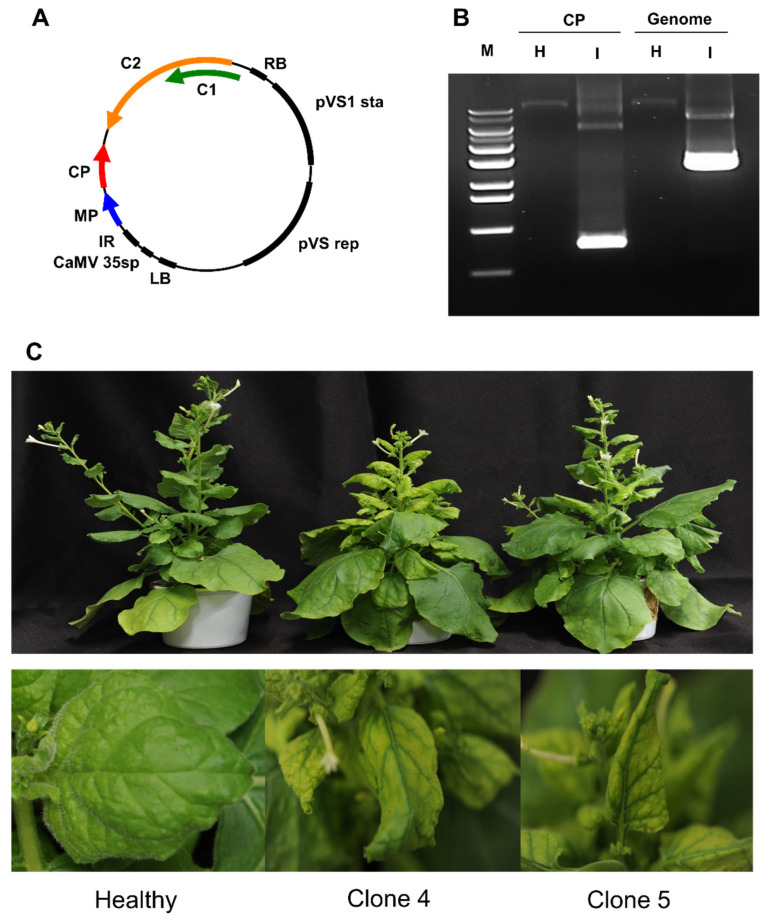
Generation of infectious clones for SGVB and determination of the symptoms in *N. benthamiana.* (**A**) Schematic representation of the vector for constructing the infectious clones of SGVB. (**B**) Detection of SGVB by PCR using CP- and genome-specific primer pairs. H (healthy plants inoculated by empty vector) and I (plants inoculated by infectious clone) of SGVB. The CP and genome indicate primer pairs amplifying the CP and SGVB genome, respectively. (**C**) *N. benthamiana* plants agroinoculated by empty vector (healthy) and infectious clones (Clone 4 and Clone 5). Photographs were taken at 3 weeks post-infection.

Due to the coinfection of different viruses in the soybean sample, it was impossible for us to identify disease symptoms caused solely by SGVB. Therefore, we generated infectious clones for SGVB. For the generation of geminivirus infectious clones, two replication origins located in the IR of geminiviruses are required. In general, tandem repeats of viral genomes are constructed in the vectors. However, this method requires a multi-step assembly of the genomic fragments. Here, we developed a simple method by inserting the IR1 sequence of SGVB, followed by the linear complete genome sequence of SGVB, into the pCAMBIA vector (Figure 6A). As a result, the vector contained dimeric genomic components. The vector was transformed into E. coli DH10B and used for rub-inoculation on the soybean leaves. Three different soybean cultivars, named Buseok, Cheongja 3-ho, and Taekwang, respectively, were used for the rub-inoculation. At 30 d post-infection, none of the soybean plants showed any visible symptoms of disease caused by SGVB.

Next, the infectious clones were transformed into A. tumefaciens strain GV3101. We agroinoculated two different infectious clones on the leaves of *N. benthamiana*. At 3 weeks post-infection, we collected leaves from mock plants and the agroinoculated plants. Using CP- and genome-specific primers, we conducted a PCR to confirm the infection with SGVB. Only the expected PCR products were amplified from the agroinoculated plants (Figure 6B). We also found that two different infectious clones (Clones 4 and 5) caused symptoms of viral disease, such as chlorosis and leaf curling, with a latent infection in the infected *N. benthamiana* plants (Figure 6C).

## 4. Discussion

In this study, we identified a novel DNA virus in soybean through RNA-sequencing. The identified SGVB was a kind of geminivirus with a single-stranded DNA genome, and we only identified a partial sequence of SGVB through RNA-sequencing. In fact, it is no longer a surprise that a virus with a single-stranded DNA genome can be identified by next-generation sequencing (NGS) or by high-throughput sequencing. For example, RNA-sequencing using mRNA libraries has previously revealed two independent pepper viromes, in which a geminivirus was redundantly presented [24]. Furthermore, a novel geminivirus in tomato and cleome plants, a novel grapevine geminivirus A in grapevine, and a novel apple geminivirus in apple have been identified using NGS techniques [25,26,27]. Similarly, viroids with a single-stranded circular RNA genome have also been identified [28,29]. It is likely that NGS techniques are very useful for identifying novel viral pathogens regardless of their genome type.

RNA-sequencing followed by de novo assembly approaches is a very powerful tool for obtaining complete or nearly complete genomes of novel RNA viruses. As compared to other RNA viruses, a complete or nearly complete genome of a DNA virus often cannot be obtained by RNA-sequencing, as we have demonstrated in a previous study [24].

In the case of DNA viruses with a circular genome, the RCA method has been widely employed [30,31,32]. The RCA method does not require specific primers for the target virus, and it uses the bacteriophage phi29 DNA polymerase [32]. However, a circular genome of a DNA virus cannot be successfully amplified using the RCA method when the viral material is limited. To overcome the limitations of the RCA method, several previous studies have conducted a PCR using newly designed back-to-back primers to obtain a complete genome of a novel geminivirus [33,34]. In this study, we used two different approaches: the PCR with back-to-back primers and the RCA method. Both methods successfully amplified the complete genome of SGVB.

Phylogenetic analyses and BLAST results showed that SGVB could be one of the mastreviruses. Moreover, the pairwise sequence alignment with the complete genome of SGVB demonstrated that the nucleotide identity between SGVB and the CpCDV isolate CpCDV_H_SD_SD180_2013 was 71.34%, making it the closest virus to SGVB. According to the criteria for species demarcation for the genus *Mastrevirus* in ICTV, nucleotide sequence identities that are less than 78% when compared to the isolates of any recognized species are considered to belong to a distinct species. Thus, the SGVB isolate Kong could be a novel species of the genus *Mastrevirus*. This is unlikely, as SGVB contains three IRs, whereas typical mastreviruses contain two IRs, such as a long IR and a short IR. As we expected, SGVB has a nonanucleotide origin of replication (5′–TAATATTAC–3′) in the stem-loop structure, which is highly conserved in the genome of geminiviruses.

As compared to other Asian countries, only a few species of geminiviruses have been reported in Korea to date, with the most common being the tomato yellow leaf curl virus (TYLCV) and the sweet potato leaf curl virus (SPLCV) [35,36]. TYLCV usually infects tomato, while SPLCV infects sweet potato. Both viruses are members of the genus *Begomovirus* and are usually transmitted by the whitefly vector, *Bemisia tabaci*. In addition, SPSV1 in the genus *Mastrevirus* has often been identified in sweet potato in Korea [35]. Although SGVB has been identified in this study, SGVB might not be a common DNA virus in Korea. It is known that mastreviruses are usually transmitted by specific leafhoppers; however, we did not obtain any information associated with an insect vector for SGVB.

The soybean sample in which SGVB was identified was co-infected by other RNA viruses, including SMV, SYMMV, and SYCMV. Therefore, it was impossible for us to observe specific disease symptoms caused only by SGVB. To reveal the disease symptoms caused by SGVB, we generated infectious clones for SGVB, since the cloning of the full-length genome of a geminivirus is easy due to its small genome size. It is known that more than one copy of a viral genome can efficiently promote the infectivity of the genomic components. In general, tandem repeats of viral genomes, which comprise two origins of replication (V-*ori*), are cloned in vectors [37]. Alternatively, PCR and Gibson assembly (GA) methods were used to generate infectious clones of the bipartite *Begomovirus* [38]. In this study, we simply added a copy of the IR1 region of SGVB, comparing the V-*ori* in addition to the full-length genome of SGVB. This approach resulted in the duplication of the V-*ori* and successfully induced the infectivity of SGVB in *N. benthamiana* via agrobacterium-mediated inoculation. Thus, we suppose that the duplication of the V-*ori* might be sufficient to efficiently induce the infectivity of geminiviruses.

Unexpectedly, we did not observe any visible symptoms of disease caused by SGVB in the three rub-inoculated soybean (*Glycine max* L.) cultivars, although SGVB was identified in soybean. The three soybean cultivars examined in the present study were the most common soybean cultivars in the region where SGVB was identified. As shown by the phylogenetic analyses and the BLAST search, SGVB was closely related to known CpCDV, which infects chickpea. It is highly likely that soybean is not a proper host for SGVB. To conform to Koch’s postulates, we examined the infectivity and disease symptoms caused by SGVB in *N. benthamiana,* which is susceptible to many plant viruses. The inoculated *N. benthamiana* plant showed typical viral disease symptoms caused by a geminivirus demonstrating the pathogenicity of SGVB.

In conclusion, we identified a novel geminivirus in soybean referred to as SGVB. The genome of SGVB comprises four different viral ORFs and three IRs. IR1 includes a nonanucleotide origin of replication in the stem-loop structure. Phylogenetic and BLAST analyses demonstrated that SGVB could be a novel virus belonging to the genus *Mastrevirus* in the family *Geminiviridae*. We generated infectious clones for SGVB by adding a copy of the IR1 region of SGVB, comparing the V-*ori* in addition to the full-length genome of SGVB. Using the infectious clones, we observed viral disease symptoms in the inoculated *N. benthamiana* plants, while none of the inoculated soybean plants showed any visible disease symptoms. This study provides the complete genome sequence and infectious clones of a novel *Mastrevirus* referred to as SGVB isolated from soybean in Korea.

## Figures and Tables

**Table 1:** Information on four proteins and three intergenic regions (IRs) in the SGVB genome.

Region	Position	Size (aa) or (nt)	Homologous Protein	Homologous Virus	Accession No.	Coverage	Identity
MP	145–438 (sense)	97 aa	Movementprotein	CpCDV	AIY33051.1	92%	68.89%
CP	705–1184 (sense)	159 aa	Coatprotein	Chickpea redleaf virus 2	QDA77210.1	100%	89.94%
C1	2005–2496 (antisense)	163 aa	RepB	CpCDV	AHF52852.1	69%	71.93%
C2	1550–2431 (antisense)	138 aa	RepA	CpCDV	AMN14229.1	100%	77.47%
IR1	2497–2616, 1–144	264 nt					
IR2	439–704	266 nt					
IR3	1185–1449	265 nt					

Each ORF was predicted by ORFfinder. Each SGVB protein was subjected to a BLASTP search. The best-matched protein for each viral protein is listed with the sequence coverage and protein identity.

## Data Availability

The full-length genome of the SGVB isolate Kong has been deposited in GenBank with the accession number MH428830.

## Supplementary Materials

The following are available online at https://www.mdpi.com/article/10.3390/plants11131768/s1, Figure S1: Pairwise sequence alignment of full-length SGVB and CpCDV isolate CpCDV_H_SD_SD180_2013.
