# Supplementary material for: Complete Genome Sequence of a Novel Monopartite Mastrevirus, Soybean Geminivirus B, Isolated from Soybean (Glycine max (L.) Merrill)
Source: Plants (Basel). 2022 Jul 3;11(13):1768. doi: 10.3390/plants11131768 (PMC9269612; doi:10.3390/plants11131768)
Supplement: Supplementary file 1 [file plants-11-01768-s001.zip › Figure S1.pdf]

```
SGVB_genome  TAATATTACGGCGTGGCCCACCTTATCGCGAGCCCTTTAGGGCGAGCGGTTTAGATTGAT   (1-60)
KM229842.1   ............................................................

SGVB_genome  TTGAAATTTAAAAAGTAAAGTTAGGCGGGAAAGTGATTGTCGCATTCACACTTTATATAA   (70-120)
KM229842.1   .....AT....................GGAACGT...............ATTTTGTATCA

SGVB_genome  AGGGGCACTCAGAGTATACCGTATATGGAGCGTCTATTATACCAGGTATTCCCCGTAAAT   (130-180)
KM229842.1   ...GGTATT.....TCCTTCGGATACGAA.......TTAT...................T

SGVB_genome  TATACGCCATCTTACGAGTCTGAGAGTTATACTGCTCCGTCGCAGGCTTCAAGTAACAAC   (190-240)
KM229842.1   CATACG..ATCCTCCT..CCAGTGAG...TGCTGCTCCA...CAGGCATCATCTCAAACG

SGVB_genome  GAGTTCGGGAAAGTTGTCATCGCTTTGGTTATTGTTCTTGTCGCTGTAGGTGTTGTTTAT   (250-300)
KM229842.1   GATTTTGGTAAGGTTGTGATCGCCTTGGTGGTTATTCTGGTTAGTGTTGGTGTGTTCTAT

SGVB_genome  TTAGCTTATTCTTTATTTTTAAAAGATTGTATTTTGTTATTTAAAGCCAAAAAGCAGAGG   (310-360)
KM229842.1   CTGGCCTATACCTTGTTCTTGAAGGATTGTATCCTCTTGTTCAAGGCGAAGAAACAGAGA

SGVB_genome  ACAACGACCGAAATAGGGTTTGGAAATACCCCTGCT..AGGCCCGG....TTCTCAACCT   (370-410)
KM229842.1   ACCACTACCGAAATCGGGTTTGGACAAACCCCGGCTCGAAATCAGGATCATCCTCAACC.

SGVB_genome  GCACAAAATGGTGGGACCCTTTAAAGGTAACGTCTACAGGAGGAAGAGAGGAAGGAACGC   (420-470)
KM229842.1   GTA.GGCGGGGTGCATCATGTCGACTGTGACGT....GGGAAAAGAAGCGGAAGCGTAGC

SGVB_genome  TAGGGTTTATGATGCACTGGGTGTTAACTCCCAACGGGATCTGAGTCAACGCTCCATCAA   (480-530)
KM229842.1   GGAGGAGAATCAAGGGCGAAGAGT.........AGGGCATCTGGTTC..CTATGTGCCTA

SGVB_genome  GGTCTATTTCCAATAGACGTGAATCCTTGCAGGTTGCAACGTTCACTTGGACCAGTTCTG   (540-590)
KM229842.1   GGTCCGTGTCTTCCAGGAGGGAATCGTTGCAGGTGGCAACCTTTTCCTGGACGAGTTCTG

SGVB_genome  GTGCGGGTGTAAAGTTTTCTGCTGGTGGGGCTGCTTTTCTGTTAAGCAATTTCCCACAGG   (600-650)
KM229842.1   GTTCGGGTATAAAGTTTTCTGCTGGTGGTGCTGCCTACCTTGTGAGTAATTTTCCACAGG

SGVB_genome  GTGCCAATGATAACTGCCGTCACACCAACAAGACAATCTTGTACAAGTTAATGTGCAAGA   (660-710)
KM229842.1   GTGCCAACGATAACTGTCGTCACACCAACAAGACCGTGCTATATAAATTCATGGCTAAGA
```

```
             720       730       740       750       760       770
SGVB_genome  ACAGTGTGTATGTGGATGCCTCTCACTATCCGAAGGTTTTCCGGTGCCCCTGTACGTTTT
KM229842.1   ATACTGTGTATTTGGACCCTAGTCACTATTCGAAGGTGTTCAGGTGCCCTTTTACTTTTT

             780       790       800       810       820       830
SGVB_genome  GGTTGGTGTATGACAAATCACCTGGGTCTAGTATTCCTAGTACTGGGGATATTTTCGAAG
KM229842.1   GGCTGGTGTACGACAAGGCTCCTGGTGCTAATGTTCCGAGTACGAGTGACATATTTGAAG

             840       850       860       870       880       890
SGVB_genome  GTCCTTCGTTGTTTCCTAATAACCCGAAGGTGTGGTCAGTTTCAAGGGCAGCATGTCATC
KM229842.1   GCCCATCTCTGTTTCCGAACAATCCTTGGACGTGGACTGTGTCTAGGGCTGCATGCCATC

             900       910       920       930       940       950
SGVB_genome  GCTTTGTGGTGAAGAAGACGTGGACCGTG.ATGCTTGAGTGTAACGGTGTTGACCCAAGC
KM229842.1   GTTTTGTTGTTAAAAAAACGTGGAC.ATGTATAGTGGAGAGCAATGGTATTGACCCAGGA

             960       970       980       990       1000      1010
SGVB_genome  AAGGCGCAGAGTGCAAGTTATTATGGGCCTGGTCCGTGTAACCAGGTGAAGTTTGTCTCC
KM229842.1   AAGGCCCAGGGTTCTACATATTATGGGCCTGGTCCTTGTAATCAGATTAAGTCCTGTAAT

             1020      1030      1040      1050      1060      1070
SGVB_genome  AAGTTCTTCAAGAGATTGGGTGTTAGCACTGAGTGGAAGAATAGTTCCACGGGTGATGTA
KM229842.1   AAGTTCTTTAAGAGATTGGGTGTGTCAACGGAGTGGAAAAATAGTGCAACGGGGGATGTT

             1080      1090      1100      1110      1120      1130
SGVB_genome  GGTGATATAAAAGAAGGGGCAATGTACATTGTTGTTGCCCCTTCGCAGAAGTCTGATGTG
KM229842.1   GGTGATATAAAGGAAGGAGCCCTTTACATTGTTGGTGCTCCTTCCCAGAAGTCTGATGTA

             1140      1150      1160      1170      1180      1190
SGVB_genome  TATGTAAATGGTTATTTCCGAGTGTACTTCAAGTCCGTTGGTAATCAATAAAATAAGTTT
KM229842.1   TATGTAAATGGTTATTTCCGAGTGTACTTCAAGTCCGTTGGTAATCAATAAAATAT.TTT

             1200      1210      1220      1230                1240
SGVB_genome  TATTATGTTTATAATATATTGCTTCAGTATGAAGAAA........ATTACAA...GTACA
KM229842.1   TATTATGAATATGTTACAATGTGCTAGTAGATAGAAATTACATATATTACAATACACACG

             1250      1260      1270      1280      1290      1300
SGVB_genome  ACATAAACACACAAAAACACACAAAAAAACAGAAACAAAATCACACCCCAAACATATTAC
KM229842.1   AAATAAACATA.AAAAACACACCCTAAAA.AAAGGCAAAAAAACACCCCTAAAACATAAC

             1310      1320      1330      1340      1350      1360
SGVB_genome  AGTATAGTGACCCTCAGTTAGAAGGAGAGGCACGTTTAGTGACTCGACGCAACCTGAGCA
KM229842.1   ACTAGA..TACCCTCGTCT..GAGGAGAGGCACGTTCAGTGACTCGACGATTGCCGAGCA

             1370      1380      1390      1400      1410      1420
SGVB_genome  AAAAATGTTTCTCCATCACACATGTAATGGATTACACAATTAGCTTCAAAGTAGTCTTTC
KM229842.1   AAAAAAGTCTCCCCGTCATACATGTAGTGGGTGAGACAATTATCTTCAAAGTAATCCTTC
```

```
                 1430      1440      1450      1460      1470      1480
SGVB_genome  TGTTGAGATGTCATATTATGCATCCAGTCTTCGTCGTCATTGCAAATGATTATGGAAGGG
KM229842.1   TGTTGACTTGACATTGAGAGCATCCAGTCTTCGTCGGGATTACAAAGAATTATTGAAGGG

                 1490      1500      1510      1520      1530      1540
SGVB_genome  ATCCCACCTTTTATTTTCTTCTTTTTTCCATATTTAGGGTTGACAGTGTAGTCATACTGG
KM229842.1   ATCCCACCTTTTATTTTCTTCTTTTTTCCATATTTAGGGTTGACAGTAAAATCAGACTGG

                 1550      1560      1570      1580      1590      1600
SGVB_genome  CAACCTATTAATTGCTTCCAGAGTGGGACGAATTTGAAGGGGATGTCGTCAATGACGTTG
KM229842.1   CAACCTATTAATTGCTTCCACAATGGGACGAACTTGAAGGGGATGTCGTCAATGACGTTG

                 1610      1620      1630      1640      1650      1660
SGVB_genome  TAGGTGGCATGGTCGTCGTAGGTGGTGTAGTCGATGGTTCCGTTCCAGTAATTGTGTCGT
KM229842.1   TACGTCGCATGTTCGTCGTATGTTGTGAAGTCGATGGTCCCATTCCAGTAGTTGTGTCGT

                 1670      1680      1690      1700      1710      1720
SGVB_genome  CCGAGGCTTCTTGCCCATGTTGTCTTGCCGGTTCTGGTTGGTCCGCAGATGTAGAGGGAG
KM229842.1   CCGAGGCTTCTAGCCCAGGTGGTCTTACCGGTACGAGTTGGTCCGCAGATGTAGAGGCTG

                 1730      1740      1750      1760      1770      1780
SGVB_genome  GGGTGTCTGGTTCTAGTCCCTCCATCGTCCTGGTTAAATCAGCCATCCATTCAAGGTCAG
KM229842.1   GGTGATCTGGTTCCAGTCCTTCTCTCGTCCTGGTTATATCGGCCATCCATTCAAGGTCAG

                 1790      1800      1810      1820      1830      1840
SGVB_genome  CTTGGGCTTCACTATATGGGACTGGATGTATATATGTGTAAGCGTCAATACTTACATGAT
KM229842.1   ATTGAGCTTGATGATATGAAACTGGATGTACGAGGGTATAGGCTTCGATGCTTACGCTGT

                 1850      1860      1870      1880      1890      1900
SGVB_genome  ATAGATGTTGTTCCCTCCA.AGTGGCTAGGTCTTCGTGGCAGCGAAGATCTTCAGTGC.T
KM229842.1   ATAGGTGTGTGTCTCTCCATAGTTG.TAAGTCTTCGTGGCAGCGAAGATCTG.ACTCCGT

                    1910      1920      1930      1940      1950      1960
SGVB_genome  GATGGAAGGTACATATGTTTCTGGTTGTGGTGGAAAGAGCCTGTTTGCTGAGTATTCTAG
KM229842.1   GAAGGGCGACACATATGGTTCAGGTTGTGGAGGGAATAATTTGTTGGCTGAATATTCCAA

                    1970      1980      1990      2000      2010      2020
SGVB_genome  CCACTGTAATTTAGTGGCCCATTCATGAGGAAATTGTTCTTTGATCATCTCGAGATATTC
KM229842.1   CCATTGAAGCTTTGTTGCCCATTCATGAGGAAATTCTTCTTTGATCATGTCAAGATATTC

                    2030      2040      2050      2060      2070      2080
SGVB_genome  CTCCTTAGATGTTGCAGTCTGGATAATAGTTCTCCATCGTGCATCAGATTTTGAAGGAGA
KM229842.1   CTCCTTAGACGTTGCAGTCTGGATAATAGTTCTCCATCGTGCGTCAGATTTGCGAGGAGA

                    2090      2100      2110      2120      2130      2140
SGVB_genome  GACTCGATGATCTTTGAAGTCTCCTCTGGTTTTGACATCTCCGTCCTTAGAAATGTATTC
KM229842.1   GACTTTATGATCTCGGAAATCTCCTCTGGTCTTAATATCTCCGTCCTTTGATATGTAATC
```

```
                      2150      2160      2170      2180      2190      2200
SGVB_genome    AAGGACTTGTTTAGAGTTTCTAGCTGGTTGGATATTAGGGTGATTTCCTTCAAAATCGAA
KM229842.1     AAGGACTTGTTTAGAGTTTCTAGCTGGCTGGATATTAGGGTGATTTCCTTCAAAATCGAA

                      2210      2220      2230      2240      2250      2260
SGVB_genome    AAAAGAAGAATCCCTAATACAAGGTTTTTTATCAAGTTGAATAAGAGCGTGGTAATGGGT
KM229842.1     AAAAGAAGGATCCCTAATGCAAGGTTTTTTATCAAGTTGGATAAGAGCATGATAGTGGGT

                      2270      2280      2290      2300      2310      2320
SGVB_genome    AGTGCCATCCTGATGAAATTCAGTAGCAATAGCAAGGTAATAAATAATAAAAGGTGTAAG
KM229842.1     AGTGCCATCTTGATGAAGCTCAGTAGCAAGACCAAGAAAGAAAATAAGAAAAGGTGTGAG

                      2330      2340      2350      2360      2370      2380
SGVB_genome    CTTTTCCCAGAGAAACTCGAAGAGTTCATCTCTCTGGGAAGAACATTTGGGGTATGTGAG
KM229842.1     TTTCTCCCAGAGAAACTGGAACAGATCATCTCTTTGAGATGAGCATTTGGGATAGGTAAG

                      2390      2400      2410      2420      2430      2440
SGVB_genome    GAAAACATATTTTGATTGAAGTCTGAATGAGGCGTTGGGTGTTCGAGGCATATTGAAGTA
KM229842.1     GAAAACATATTTAGATTGGAGTCTGAA...GTTCTTGTTAGCAGAAGGCATGTT......

                      2450      2460      2470        2480      2490
SGVB_genome    AGTTGTGACTCCATACAGTGATCCCCAAACTCTAT..TATAACAGGGCGTGGAGGCATGG
KM229842.1     .GTTGTGACTCCGAGGGGTTG.CCACAAACTCTATCTTATAACAGG.CGTGGAGGCATGG

                2500      2510      2520      2530      2540      2550
SGVB_genome    AGGCAAGGGCATTGTGGTAATTTGGAAAGTAAGTGGAATTGCACGTGGCATTTACTTAGA
KM229842.1     AGGCAAGGGTATTTTTGTCATTTGGATATATAGTGGAAAATGACGT..CATTTACTTAAG

                2560                          2570                    2580
SGVB_genome    AATTAAG.......TT..........TTAAAA..................CTT.TCGC.A
KM229842.1     CGTTAAGGGGGGAATCCACGCCGAAATTAATATTACCGGCGTGGTCCCCCCTTATCGCGA

                               2590         2600          2610
SGVB_genome    GAA....AGTGC......CCGGGAT..AAGGTGGG....TCCACGCCCATT.........
KM229842.1     GCACTTTAGTGCGAGCGGTCCAGATTTAAAGTGGTAAATTTCCGCCCACTAGGGTTTGT

SGVB_genome    .............................................
KM229842.1     CTTGCTTTTGCTTTATAAAAGGCGCACTAATGTATAGACCGTAGA
```

Figure S1. Pairwise sequence alignment of full-length SGVB and CpCDV isolate CpCDV_H_SD_SD180_2013.
